# Living with Pompe disease: results from a qualitative interview study with children and adolescents and their caregivers

**DOI:** 10.1186/s13023-024-03368-7

**Published:** 2024-09-28

**Authors:** Moritz Ilan Truninger, Helene Werner, Markus Andreas Landolt, Andreas Hahn, Julia B. Hennermann, Florian B. Lagler, Dorothea Möslinger, Charlotte Pfrimmer, Marianne Rohrbach, Martina Huemer

**Affiliations:** 1 Division of Metabolism, Children’s Research Centre, University Children’s Hospital Zurich, University of Zurich, Steinwiesstrasse 75, Zürich, 8032 Switzerland; 2 Department of Psychosomatics and Psychiatry, University Children’s Hospital Zurich, Steinwiesstrasse 75, Zurich, 8032 Switzerland; 3 Division of Child and Adolescent Health Psychology, Department of Psychology, University of Zurich, Binzmühlestrasse 14, Box 8, Zürich, 8050 Switzerland; 4 Department of Child Neurology, Justus-Liebig-University Gießen, Gießen, Germany; 5 Villa Metabolica, Center for Pediatric and Adolescent Medicine, University Medical Center Mainz, Mainz, Germany; 6 Institute for Inherited Metabolic Diseases, Department of Pediatrics, Paracelsus Medical University, Salzburg, Austria; 7 Department of Paediatrics and Adolescent Medicine, Medical University of Vienna, Vienna, Austria; 8 Department of Paediatrics, LKH Bregenz, Bregenz, 6900 Austria; 9 Competence Area Healthcare and Nursing, Vorarlberg University of Applied Sciences, Hochschulstr.1, Dornbirn, 6850 Austria

**Keywords:** Health-related quality of life, Functioning, disability, and health, Pediatric patients, Qualitative analysis, Content analysis, Concept elicitation interviews, Content validity, Lysosomal storage diseases, Inborn errors of metabolism, Muscle weakness

## Abstract

**Background:**

Children and adolescents with Pompe disease (PD) face chronic and progressive myopathy requiring time-intensive enzyme replacement therapy (ERT). Little is known about their perspectives on the disease and its treatment. This study explored their perceptions of disease symptoms and functioning status, and more subjective feelings about the impacts on their lives as part of developing a disease-specific questionnaire.

**Methods:**

Eleven pediatric patients aged 8–18 years and 26 caregivers from six children’s hospitals in Germany, Austria, and Switzerland underwent semi-structured interviews. Data were recorded, transcribed using MAXQDA software, and analyzed using qualitative content analysis. A system of meaningful categories was developed.

**Results:**

Sixteen main categories were derived across four major thematic areas: perceptions of symptoms and limitations, experiences to do with the biopsychosocial impact of PD, treatment experiences, and general emotional well-being/burden. Participants demonstrated broad heterogeneity in symptom perceptions such as muscle weakness, breathing difficulties, pain, and fatigue. Emotional appraisals of limitations were not directly proportional to their severity, and even comparatively minor impairments were often experienced as highly frustrating, particularly for social reasons. The main psychosocial topics were social exclusion vs. inclusion and experiences to do with having a disease. The main finding regarding treatment was that switching ERT from hospital to home was widely viewed as a huge relief, reducing the impact on daily life and the burden of infusions. Emotional well-being ranged from not burdened to very happy in most children and adolescents, including the most severely affected.

**Conclusion:**

This study provided qualitative insights into the perceptions and experiences of pediatric PD patients. Interestingly, biopsychosocial burden was not directly related to disease severity, and tailored psychosocial support could improve health-related quality of life. The present findings ensure the content validity of a novel questionnaire to be tested as a screening tool to identify patients in need of such support.

## Background

Pompe disease (PD; OMIM♯232300; Online Mendelian Inheritance in Man, OMIM^®^), also known as glycogen storage disease type II and acid maltase deficiency, is a very rare, progressive, and often debilitating metabolic myopathy. Inheritance is autosomal recessive: pathogenic variants in the *GAA* gene cause deficient activity of the enzyme acid alpha-glucosidase, which breaks down lysosomal glycogen. The age of onset is variable, and phenotypic variance is large, ranging from the classic and rapidly progressive infantile-onset type (IOPD) to the attenuated late-onset type (LOPD) [1]. Both types are characterized by progressive muscle weakness, which often leads to limitations in motor and respiratory functions. IOPD patients usually have very severe muscular hypotonia and the heart is also affected in terms of a cardiomyopathy. Untreated, IOPD usually leads to cardiorespiratory failure within the first years of life [2, 3]. Enzyme replacement therapy (ERT), approved in 2006, reduces mortality effectively in IOPD [4] and improves physical and respiratory function in LOPD [5]. However, ERT is time-consuming, costly and does alleviate but not cure the disease. Patients continue to suffer progression of symptoms [6, 7]. Under ERT, a new IOPD phenotype has emerged, including long-term survivors who may achieve developmental milestones like walking independently and/or sitting unassisted [4, 8]. Still, most IOPD patients experience secondary decline during childhood and the loss of achieved milestones. Also, severe breathing difficulties requiring invasive ventilation, swallowing difficulties and need for nasogastric tube or gastrostomy, as well as hearing difficulties are common in IOPD [6, 9]. Children and adolescents with LOPD are comparatively less affected in terms of both motor and respiratory function. However, the phenotypic spectrum is wide, ranging from asymptomatic at time of diagnosis to patients needing walking aids and/or ventilation at some point even with treatment [5, 10]. Both types may experience other symptoms including pain, fatigue, speech difficulties, and gastrointestinal complaints [4, 11]. Given the chronic nature of PD and the lifelong need for medical care, it is important to understand how best to support patients and families. Insight into subjective outcomes such as health-related quality of life (HRQoL) are crucial for this purpose [12].

Many definitions of HRQoL emphasize that it is a subjective and multidimensional construct about the biopsychosocial impact of disease and treatment [13, 14]. However, HRQoL is often not clearly distinguished from related concepts, in particular, from functioning, disability, and health (FDH), also termed health or functioning status [15, 16]. Cieza and colleagues [15, 17, 18] have provided helpful clarification by defining HRQoL as subjective feelings, emotions, and appraisals about health-related domains (e.g., satisfaction with physical abilities), whereas FDH includes subjective self-reports on objective health-related facts (e.g., statements about the ability to walk for a long time). In this paper, we adopt this distinction and refer to FDH-related aspects as “perception” and HRQoL-related aspects as “experience.”

Efforts have been made to examine disease-specific FDH and HRQoL through qualitative studies with adult PD patients and experts and the development of self-report questionnaires [19–22]. However, very little is known about the health perceptions and appraisals of children and adolescents with PD. Today, only one disease-specific proxy report is available for this age group, and it focuses solely on mobility and self-care from an FDH perspective (Pompe-PEDI; [23]). Our research group is currently developing the first disease-specific questionnaire for pediatric patients with PD that covers FDH and HRQoL through self- and proxy-report (manuscript submitted for publication by our research group). Creating items with high content validity requires concept elicitation interviews with patients to identify meaningful topics [24, 25]. Such qualitative approaches can facilitate a better understanding of the burdens and needs of the patient population and provide a starting point for improving patient support, clinical care, and ultimately their HRQoL.

This paper presents a qualitative content analysis of interviews with children and adolescents with PD and their caregivers. The study identifies key topics of PD-specific FDH and HRQoL and thus provides insights into their lives. We explore four major thematic areas: (1) perceptions of symptoms and related limitations, (2) experiences to do with the biopsychosocial impact of PD on their lives, (3) experiences to do with treatment, and (4) general emotional well-being/burden.

## Methods

### Study design, subject recruitment, and participants

This qualitative study is international and multicenter in scope. It was conducted in full accordance with the Declaration of Helsinki and approved by the review boards of the participating institutions in Bregenz, Giessen, Mainz, Salzburg, Vienna, and Zurich. Local metabolic physicians invited patients aged 8 to 18 years with PD and parents of patients older than 3 years to participate either by phone or during regular appointments. Exclusion criteria were an insufficient command of German and, for patients only, the incapacity to follow the study procedures (e.g., due to severely reduced health status).

Nineteen families participated in the study: 11 children and adolescents, 17 mothers, and nine fathers. In two families, only the patient was interviewed; in four families, the patient and the mother; and in five families, the patient and both parents. In four families, only the mothers were interviewed; in another four families, both parents participated in interviews.

### Materials and procedures

#### Sociodemographic and medical characteristics

Informed consent was obtained from participants before recording basic information on sociodemographics (sex, age, children’s mother tongue, parents’ country of birth), PD type (IOPD vs. LOPD), age at diagnosis, walking capacity, respiratory support, and ERT (location, frequency).

#### Qualitative interviews

**Fig. 1:**
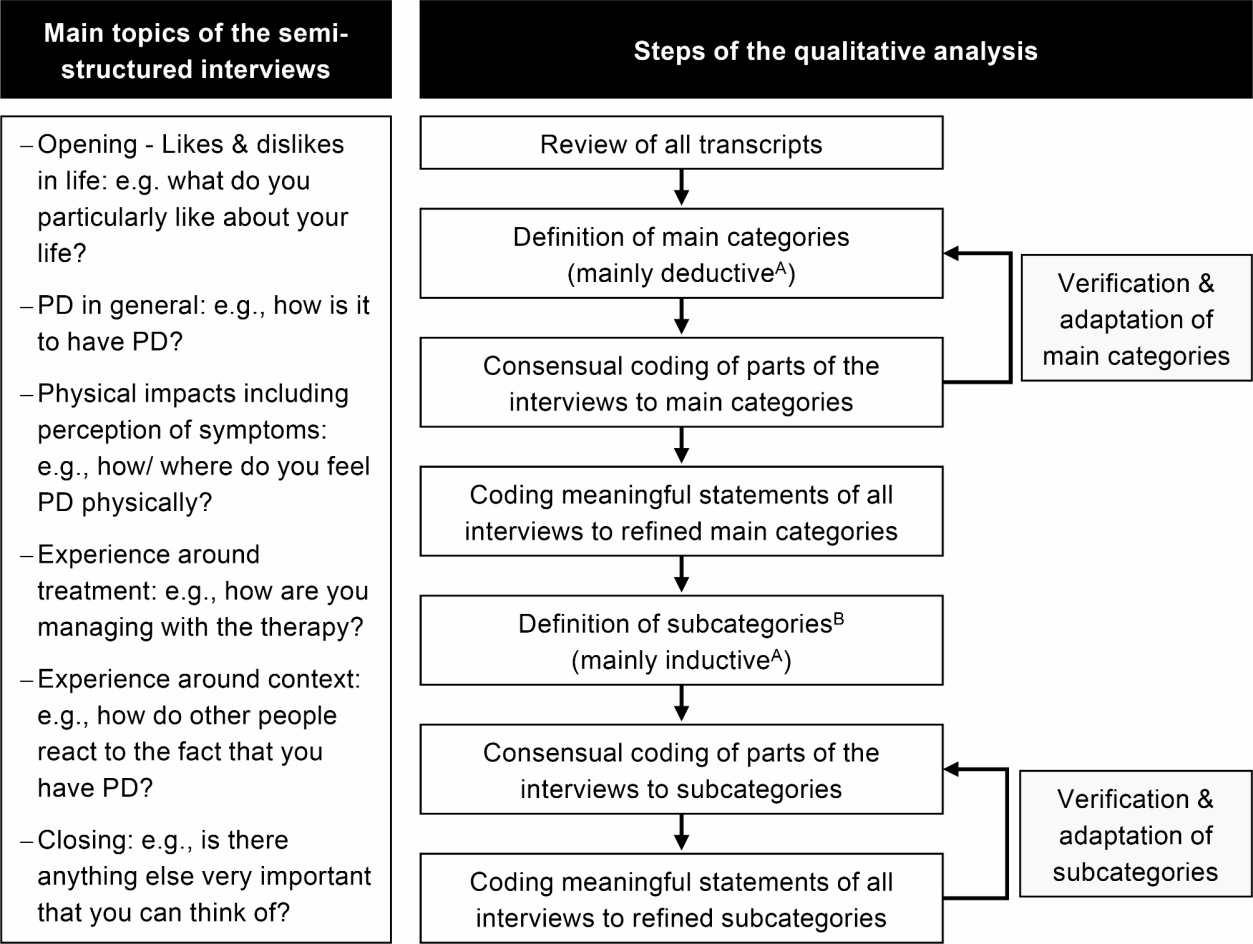
Main interview topics and analysis procedure based on Kuckartz [29] Note: Figure design was adapted from [27]. Coding remarks: Only meaningful statements were coded, that is, statements directly relevant to the four major thematic areas (e.g., parent-related experiences were not coded); Some statements addressed past perceptions or experiences. These were coded the same as those related to the present; A transcript passage could be assigned to multiple categories if it contained meaningful statements for more than one category (as outlined in [29]. ^A^ A mixed deductive-inductive approach was applied, combining prior knowledge (deductive; e.g., literature on PD and HRQoL) and observations from the interviews (inductive) to derive categories. ^B^ Subcategories were only derived for a given main category, if clearly distinct sub-aspects were described

The interviews were conducted by the first author (MIT), a trained moderator with a background in psychology. Because the study started in 2021 during the COVID-19 pandemic, one-on-one online interviews were arranged for all participants. For one family, an adolescent patient and mother, online interviews were not possible, so they were interviewed at their home. In four patient interviews, a caregiver was present, primarily for assistance due to speech difficulties. In one case, the father of the interviewee repeatedly interrupted the conversation, leading to the exclusion of the corresponding data. One pair of parents wanted to be interviewed together. All interviews followed a semi-structured format. The procedure was documented in a manual, based on similar previous projects for other diseases [26, 27]. It underwent revisions and adaptations following feedback from several experts, including three physicians specialized in PD, a home care nurse with experience in ERT, and an adult patient with PD. The final manual contained various open questions for several topics (see Fig. 1, left side). In addition, it included possible follow-up questions for very briefly answered questions such as “can you describe this?” and “how do you feel when this happens?” Questions for caregivers were rephrased in parallel (e.g., “what bothers your child most about having PD?”).

#### Transcription & qualitative analysis

Transcription and thematic qualitative content analysis were conducted with MAXQDA software [28] following established procedures [29, 30]. Interviews were conducted in German and transcribed verbatim by three student assistants. Statements presented in the results section of this paper have been translated into English and condensed for brevity. Content analysis was led by MIT, with support from four additional student assistants. To ensure consistency and shared understanding, assistants were briefed on the project’s aims, methods, and literature covering PD [4, 7, 19, 22, 31–35], HRQoL, and related concepts [15–17, 36–40].

A set of main categories and subcategories was derived in several analytical steps (see Fig. 1). Initially, two coders collaboratively derived preliminary main categories from the four major thematic areas: (1) perceptions of symptoms and related limitations (FDH): reports of objective states of symptoms and associated impairments in daily activities; (2) experiences to do with the impact of PD (HRQoL): subjective feelings and appraisals around the biopsychosocial impact of PD on patients’ lives; (3) experiences to do with treatment (HRQoL): subjective views of treatment and associated challenges; and (4) general emotional well-being/ burden: reports of overall emotional state. Afterward, coders independently coded parts of the interviews to main categories and then compared their transcripts (i.e., consensual coding procedure). Disagreements were discussed until consensus was reached and category definitions were specified. Subsequently, four coders assigned all meaningful statements from the transcripts to the refined main categories. Any coding uncertainties were resolved through collaborative decision-making. This process was repeated to define and code subcategories by three coders.

#### Preparation of results

After coding, statements within each category were carefully reviewed to identify key points, and the frequency of categories across cases was determined. Each case represented the data for one patient derived from one to three interviews. The number of statements in each category was evaluated for each case. Category frequency was calculated by dividing the number of cases with statements in that category by the total number of cases.

## Results

### Sample characteristics

The mean age of the patients from the 19 families was 12.0 years (SD = 5.2); 36.8% were female, 89.5% spoke German as mother tongue, and 36.8% had IOPD. Mean age at diagnosis was 4.0 years (SD = 4.5). Three patients were unable to walk (15.8%) and two required a walker or other assistance (10.5%). Four patients required breathing support (21.1%; *n* = 1 tracheostoma; *n* = 3 breathing masks). Table 1 provides an overview of patients from the 19 families, including examples of current symptoms/limitations reported during the interviews. All patients received ERT (73.7% biweekly, 26.3% weekly). Eighteen of them (94.7%) had switched to infusions either at home (73.7%) or in other locations (e.g., school; 21.1%). The mean age of the 17 mothers was 43.1 years (SD = 7.1; one was born abroad). The mean age of the nine fathers was 45.2 (SD = 6.9; none was born abroad).

**Table 1:** Overview of the characteristics of the pediatric patients from the 19 families for whom interview data was analyzed

Family Nr.	Interviewee	Age (yrs)	PD type	Age at diagnosis (yrs)	Mobility support required	Breathing support required	Examples of symptoms/limitations reported during interviews
1	pt, mth	> 16	LOPD	1–2			Slight difficulties walking (endurance, going uphill, falling)
2	pt, mth	12–15	IOPD	< 1	eWheel	tracheo	Almost no movement possible in any part of the body, considerable speech & swallowing difficulties (tube feeding sometimes required)
3	mth	> 16	LOPD	5–7	walkA		Considerable difficulties walking, inability to stand up from sitting & to climb stairs
4	pt, mth	> 16	LOPD	12–15			Slight difficulties walking (going uphill, falling) & standing for long period, pain in the back, neck, & headache
5	mth, fth	5–7	LOPD	1–2			No current symptoms/limitations reported; Past slight difficulties standing up (prior to ERT)
6	mth	5–7	IOPD	< 1^a^			Slight difficulties walking & running (speed, endurance), activity-related exhaustion, past swallowing difficulties (prior to logopedics)
7	mth, fth	5–7	IOPD	1-2^b^			Slight difficulties walking, running (speed, endurance, falling), & climbing stairs, activity-related exhaustion, slight speech difficulties; Past slight swallowing difficulties (prior to logopedics)
8	pt, mth	8–11	IOPD	1-2^b^	walkA		Considerable difficulties walking (walker needed outside)
9	mth	5–7	IOPD	< 1	mWheel	breathM (D/N)	Inability to walk or stand, difficulties driving & using a manual wheelchair, activity-related exhaustion, considerable speech & swallowing difficulties (mostly pureed food)
10	mth, fth	< 5	IOPD	< 1			Almost no current symptoms or limitations reported (slightly flabby muscles); Past considerable flabby muscles (prior to ERT)
11	mth, fth	5–7	LOPD	1–2			Slight difficulties walking (endurance, going uphill), activity-related exhaustion, slight speech difficulties, pain in the back, neck, & headache; Past slight swallowing difficulties
12	pt, mth, fth	8–11	LOPD	1–2			Slight difficulties walking, running (endurance, speed, falling, throat pain after running), & climbing stairs
13	pt, mth, fth	8–11	LOPD	8–11			Almost no current symptoms/limitations reported (slightly slower running than the average peer); Past difficulties walking & activity-related exhaustion (prior to ERT)
14	mth	8–11	LOPD	8–11			Slight difficulties running (speed, endurance), activity-related exhaustion
15	pt, mth, fth	> 16	LOPD	12–15		breathM (N)	Slight difficulties walking (speed, endurance; also due to shortness of breath & trunk, neck issues), standing for longer times, & climbing stairs, inability to run, tensions in back
16	pt, mth, fth	12–15	LOPD	8–11		breathM (N)	Slight difficulties walking (endurance, speed; also due to shortness of breath & trunk issues), standing for longer times, & climbing stairs, pain in the back
17	pt, mth, fth	8–11	IOPD	< 1	mWheel		Inability to walk or stand, pain in the neck; Past swallowing difficulties (prior to logopedics)
18	pt	> 16	LOPD	3–4			No current symptoms/limitations reported
19	pt	12–15	LOPD	< 1^a^			No current symptoms/limitations reported

Note

PD = Pompe disease, IOPD = infantile-onset PD, LOPD = late-onset PD, pt = patient, mth = mother, fth = father, eWheel = electronic wheelchair, mWheel = manual wheelchair, walkA = walking assistance, tracheo = tracheostoma, breathM = breathing mask, *N* = night, D/*N* = day and night, ERT = enzyme replacement therapy

^a^ These patients were diagnosed early due to PD-affected other family members

^b^ IOPD was diagnosed late in these patients. However, they exhibited symptoms within the first year of life, including the typical cardiomyopathy of IOPD, and have corresponding genetic mutations (according to the recruiting physician)

### Categorical system

A total of 1393 meaningful statements on PD-specific FDH or HRQoL were identified from the transcripts. First, 16 main thematic categories were derived within the four thematic areas, with one to six categories per area. Then, 12 of the 16 main categories (75%) were subcategorized.

### Area I: perception of symptoms and associated limitations

The interviews highlighted the perception of various symptoms and related difficulties, leading to the definition of six main categories with zero to ten subcategories (Table 2).

**Table 2:** Perception of symptoms and associated limitations: description of qualitatively derived categories

Main & Subcategories	Key points and examples
*Perception of…*	
1) Muscle weakness in legs, arms, & core^a^	
- Driving a wheelchair	• Difficulties driving a manual wheelchair (e.g., for long distances) or operating its brakes
- Neck muscles	• Difficulties keeping the neck upright or lifting it from a lying position
- Sitting	• Difficulties sitting upright (e.g., slumping after a while or needing to use your hands for support)
- Standing	• Inability to stand up at all, or difficulties standing for long periods of time
- Picking up & carrying things	• Difficulties picking up things from the floor and carrying them
- Walking, running & related activities	• Inability to walk or run • Special gait style or insecurity (e.g., waddling, frequent tripping, feet or legs giving way) • Reduced endurance, strength, or speed (e.g., difficulties with long or uphill walks, slower in walking or running)
- Riding a bike	• Inability or difficulties cycling (e.g., inability to ride uphill)
- Climbing stairs	• Inability or difficulties climbing stairs (e.g., being slower, needing to use the railing, leaning on thighs for support)
- Upper extremity functions	• Inability or difficulties using hands or fingers (e.g., difficulties moving fingers, writing is tiring, inability to open jars) • Difficulties lifting arms (e.g., when getting dressed)
- Other topics	• Perceiving general muscle weakness, upper body being limper, or getting muscle aches more quickly • Difficulties with sports (e.g., climbing, push-ups, or skiing) • Difficulties crawling (e.g., needing alternative crawling techniques to move around)
2) Difficulties breathing	
- At rest	• Difficulties breathing when lying (e.g., requiring respiratory support when sleeping, or otherwise being very tired in the morning, running out of breath faster the next day) • Often or always having difficulties breathing (e.g., always requiring respiratory support and still perceiving shortness of breath at times)
- During activities	• Difficulties breathing during or after activities such as walks or other exercise (e.g., running out of breath very quickly, fast breathing, or getting a sore throat)
3) Musculoskeletal pain, tension, & posture difficulties (no subcategories)	• Back, neck tensions and/or pain, ranging from rare minor discomforts to frequent severe pain^b^ • Tension-associated headache at the back of the head; sometimes extreme enough to cause vomiting • Having torso posture difficulties (e.g., troubles keeping the torso upright, having a very stiff torso and troubles turning it) • Having posture- and/or pain-related difficulties (e.g., troubles sitting, standing, walking for a longer time)
4) Fatigue/exhaustion (no subcategories)	• Often or always getting tired or exhausted due to physical activities or daily life more than healthy peers (e.g., getting tired quickly at school or being tired after school, needing more sleep or rest in daily life) • Often being very tired without apparent reason, unrelated to any activity
5) Other PD symptoms	
- Speech	• Speaking indistinctly, slurred, or softly (e.g., so that unknown people have mild to severe difficulties understanding)
- Swallowing	• Slight swallowing difficulties (e.g., difficulties with larger pieces or when having a full mouth)^c^ • Severe swallowing difficulties (e.g., inability to eat all consistencies, sometimes requiring a probe, swallowing incidents)
- Mimic	• Not having any or not as many facial expressions (e.g., lacking a regular laughing expression)
- Digestion	• Thinner to diarrhea-like bowel movements, sometimes related to occasional problems holding bowel movements, or general incontinence • Occasional constipation
- Weight, muscle mass	• Being very to extremely thin, fast weight loss or difficulties gaining weight/muscles (e.g., when training)
- Susceptibility to illness	• More frequent, prolonged, or severe respiratory infections compared to peers (e.g., need for longer recovery periods)^d^
- Other topics	• Low energy in the morning and/or morning nausea • Generally low appetite or eating small portions, but potentially more often • Hearing difficulties (e.g., requiring a hearing aid) • Other types of pain (e.g., pressure pain due to prolonged lying, discomfort due to tendon shortenings in legs and feet)
6) Symptom changes in relation to the ERT	
- Changes related to ERT start, dosage adjustments	• Continuous or sudden improvements, stabilization following ERT start or enzyme dosage increase (e.g., improved mobility, increased energy, weight gain, sudden disappearance of severe headaches within a few ERT appointments) • Worsening after a few years of ERT or due to enzyme dosage reduction (e.g., significant improvement in stair climbing ability when switching from twofold to fourfold dosage but worsening when switching back to twofold dosage)
- Changes related to individual ERT appointments	• Having fewer difficulties in the first days after infusion or having more difficulties in the days preceding the next infusion (e.g., feeling energetic right after infusion, more weakness-related difficulties such as tripping before the next infusion • Getting very tired on infusion day^e^, itching attacks on the arm, an enzyme-related allergic reaction, or having diarrhea after the infusion

Note

a-e: Potential factors influencing certain PD symptoms and associated limitations are listed (based on participants’ remarks and/or coder observations):

^a^ Muscle weakness-related difficulties may increase due to puberty-related growth in length (e.g., climbing stairs, upper extremity functions, overall muscle functioning)

^b^ Back and neck tensions and/or pain may increase during the course of the day, when doing certain activities, or due to puberty-related growth in length

^c^ Slight swallowing difficulties may improve or disappear over time, potentially due to logopedics

^d^ Susceptibility to illness may improve or disappear after early childhood

^e^ Tiredness on infusion days may be due to the procedure and/or to allergy-related drugs

1. *Muscle weakness*

Difficulties associated with muscle weakness were the disease symptoms most frequently discussed (reported in 89.5% of cases) and encompassed a wide range of impairments in daily activities, such as driving a manual wheelchair, standing, and riding a bike uphill. Ten subcategories were defined (Table 2). The most important topic was difficulties in walking and running, reported in 84.2% of cases, ranging from inability to walk to milder difficulties such as reduced running speed.

Mother of a severely disabled teenage girl, 14 years: “So, the skeletal muscles do not work at all anymore. She can only perhaps move a few fingers.”

2) *Difficulties breathing*

Breathing difficulties were reported in 36.8% of cases. These included issues at rest, such as constant need for respiratory support and difficulties breathing while lying down. Others were activity-related difficulties, such as running out of breath while walking. For some participants, breathing difficulties were the primary limiting factor in activities, more so than muscle weakness.

Teenage boy, 13 years: “When we go for longer walks, I can’t join in because my endurance is limited. . My muscles could actually still do it, but my breathing can’t.”

3) *Musculoskeletal pain, tension, & posture difficulties*

Musculoskeletal pain, tension, and posture difficulties were reported in 31.6% of cases. Back and neck tension and pain, sometimes associated with severe headaches, emerged as key issues. Frequently associated with this were postural problems such as a rigid torso and challenges in maintaining an upright position. Due to postural problems and pain, individuals also faced problems sitting or walking.

Mother of a boy, 7 years: “And what he also often has because of his posture, also simply because he is so hypotonic, is back and neck pain. He also has a lot of headaches as a result. . So that’s two or three times a week where he currently has severe headaches. . Sometimes it’s so bad that he has to vomit.”

4) *Fatigue/exhaustion*

Symptoms of fatigue were mentioned in approximately one third of cases (36.8%). Almost all statements were about activity-related exhaustion: patients got tired easily during physical activities or due to the demands of daily life.

Mother of a boy, 7 years: “You can generally say that his normal everyday life is very exhausting and makes him tired. And we always must make sure that we don’t overdo it in the morning when we’re planning activities. . So he sleeps a lot. He sleeps 12 hours a night, and he’ll even catch an hour at lunchtime if he can. But he’s not tired for no reason.”

5) *Other PD symptoms*

Most participants (89.5%) reported one or more additional symptoms. Among these, slight to severe difficulty swallowing was identified as one of the most prevalent (42.1%). In addition, some parents reported improvements or complete resolution over time and attributed this to early logopedic interventions. Frequent and/ or severe respiratory infections were also common (42.1%), with clear improvements reported after early childhood.

Father of a boy, 7 years: “We had problems with infections at the beginning. They always immediately affected the bronchial tubes, the lungs. But for a few years now, he’s seven, that’s no longer the case.

6) *Symptom changes in relation to the ERT*

Changes in symptoms related to ERT were described in 73.7% of cases. Some accounts highlighted continuous or very sudden improvements or stabilizations after starting ERT, such as disappearance of severe headache within a few appointments. Others described fluctuations between ERT appointments or on the infusion day, such as increased energy in the first days after infusion or getting tired during or after the procedure.

Adolescent girl, 18 years: “Whenever I’ve had it [ERT] again, I notice that I am simply much, much fitter. And then it actually goes on for almost two weeks. And then, yes, I realize again that it’s slowly becoming necessary again.”

### Area II: experiences to do with the impact of PD on life

Experiences to do with the impact of PD on life were grouped into five main categories, each with two to six subcategories (see Table 3).

**Table 3:** Experiences to do with the impact of PD on life: description of qualitatively derived categories

Main & Subcategories	Key points and examples
*Experiences to do with…*	
1) Specific physical aspects ^a^	
- Difficulties breathing	• Fear of suffocation or losing consciousness due to breathing difficulties ^c^
- Pain	• Slightly bothered to severe suffering and feeling very restricted when experiencing pain, depending on pain intensity
- Speech difficulties	• Bothered by them (e.g., annoyed when not understood by people) or not bothered by them anymore (e.g., accustomed to occasional misunderstanding)
- Being (very) thin/ skinny	• Bothered by it, mainly dissatisfaction with body (e.g., desire for a more muscular appearance, not wanting others to see the thinness) ^d^ or not bothered by it (e.g., acceptance of body as it is)
- Diarrhea & incontinence	• Bothered by diarrhea or worried about diarrhea-related events (e.g., not wanting to leave the house when having diarrhea, carefully thinking about what to eat to prevent such events) • Not bothered by it (e.g., not yet bothered by having to wear diapers)
- Other topics	• Not bothered by lack of facial expressions • Fear of certain food due to swallowing risk • Annoyed by frequent illness
2) Physical limitations ^b^	
- With social component	• Sad, angry, or disappointed about physically being unable to keep up with peers, siblings or to participate in activities that other people do (e.g., being the slowest in sports education or unable to take part) • Ashamed of limitations (e.g., trying to hide them) ^e^ • Not bothered by inability to keep up, participate at all times (e.g., viewing not participating in sports as a positive thing)
- Without social component	• Sad, angry, or annoyed about - Inability to do certain daily life activities at all or no longer (e.g., not able to walk (anymore), to do certain sports) - Dependency on others or on tools for tasks or activities (e.g., having to ask people to get something out of reach, requiring a scooter to get around) - Limitations making activities too exhausting (e.g., finding writing strenuous, needing to take breaks when walking) - Extensive planning required (e.g., needing to think about how to get around well when going into town) • Fear or caution due to limitations (e.g., feeling insecure when cycling) • Appreciation for what can still be done despite limitations
- Physical activities (in the context of limitations)	• Not enjoying, rejecting, or giving up physical activities where limitations play a role • Enjoying physical activities despite limitations playing a role, doing them to the best of their ability, or enjoying activities where limitations have less impact (e.g., swimming)
- Other topics	• Appreciation for not having any limitations despite the disease (e.g., feeling motivated to exercise as a result)
3) Handling of and impacts from social environment	
- Social exclusion & negative remarks	• Bothered, sad or annoyed when peers limit participation in activities due to disability (e.g., intentionally not being passed to in football games) or when people make insults or insensitive remarks about PD-related limitations • Worried about potential exclusion or insults due to the disease
- Acceptance & support from peers about the disease	• Feeling accepted by peers (e.g., fully integrated in a group of friends without PD playing a major role) • Feeling supported by peers (e.g., naturally receiving help with activities when needed)
- Other reactions of the environment	• Experiences to do with being asked about PD or having to explain the disease ranged from not minding (e.g., preferring people to ask) to ambivalent to feeling uncomfortable (e.g., annoyed when people ask, especially if repeatedly) • Bothered by being treated differently (e.g., being starred at due to a wheelchair, being pitied for the disease) • Bothered by the lack of consideration for disabilities in institutions, public places etc. (e.g., being reprimanded for using necessary equipment like e-scooters, encountering unpreparedness for disabilities on school trips) • Bothered by parents being cautious due to the disease (e.g., parents worrying a lot, forbidding things because of PD)
4) Plans & thoughts for the future	
- Future disease course	• Thinking about future symptom development (e.g., worried about worsening, hopeful for stability or improvement) • Thinking about dying, life expectancy, or the possibility of assisted suicide
- (Potential) impacts on long-term plans ^f^	• Bothered that certain plans are impossible or at least very difficult due to the disease (e.g., having to give up jobs that are physically too demanding or study or travel plans due to the ERT) • Worried about moving out from home due to your limitations or the potential impact of a genetic disease on future family plans
5) Having a disease	
- Having PD is okay	• Overall, not very bothered by having a disease, even if some aspects can be quite annoying, or not knowing life as any different anymore or yet (e.g., having PD is not a big issue in life, growing up with it as part of daily life, not yet fully aware of it ^g^) • Not defining oneself by the disease or accepting it (e.g., not feeling different from others, feeling unique in a neutral-positive manner)
- Having PD is quite burdensome	• Feeling generally sad and/or angry about having PD (e.g., it is often a big issue in life) • Defining oneself by the disease in a negative manner, feeling ashamed or not accepting it (e.g., feeling unequal to others, ashamed when standing out because of limitations or visible port, wishing it away, avoiding confrontation with it) • Bothered or frustrated by the realization that other people do not have PD and its impacts (e.g., others do not require ERT)

Note

^a^ Muscle weakness was not listed in this main category, because weakness-related experiences were manifested through experiences around physical limitations

^b^ Physical limitations were often caused by muscle weakness, but also by other symptoms

c-g: Potential factors influencing certain experiences around PD impact on life are listed (based on participants’ remarks and/or coder observations):

^c^ Fear of suffocation or losing consciousness may be especially relevant to children with a history of severe shortness of breath

^d^ Negative experiences around being thin may be especially relevant to male patients around or during puberty

^e^ Feeling ashamed of limitations may increase in the presence of unfamiliar people

^f^ Experiences to do with potential impacts on long-term plans may be especially relevant to adolescents

^g^ Lack of full awareness of having PD may be especially or exclusively relevant to young children

1. *Specific physical aspects*

More than two thirds of the patients (68.4%) had experiences, mostly negative, involving one or more specific physical aspects. Although there were relatively few statements about each aspect (percentages ranged from 15.8 to 36.8%), patients often found these experiences to be quite distressing. Pain-related experiences in particular resulted in severe suffering. Other issues included fear of suffocation, dissatisfaction with being very thin, especially among male patients around or during puberty, and worry about diarrhea-related incontinence events.

Mother of a boy, 7 years: “So the headaches have increased dramatically over the last year. And it’s very stressful because it’s now also taking away his quality of life. . We’ve already had to pick him up from kindergarten a few times because of it. And then he just lies on the couch.”

Adolescent boy, 18 years: “So I weigh very little. About 33kg. And yes, of course that bothers me a bit because I’m very thin. . for example, we went swimming recently and everyone else went swimming but me. Because I don’t like to show myself like that.”

2) *Physical limitations*

The impact of physical limitations was a very important issue for nearly all children and adolescents (89.5%). An important distinction depended on whether a social component was involved. Anger or sadness resulted from being unable to keep up with peers or to participate in social activities, regularly making even relatively minor limitations very troublesome. Experiences without a social component included frustration about being unable to perform certain activities at all or requiring extra effort or assistance. Due to the negative experience of limitations, some patients did not want to engage in physical activities in which limitations played a role, but others enjoyed the activities despite the limitations.

Mother of a boy, 7 years: “We tried soccer. . And he quickly realized that he couldn’t keep up and that was a real red flag for him. So, he categorically refuses to do things like that, where you can see straight away that something is different with him.”

Boy, 10 years, non-ambulatory: “Yes, I can’t walk; that’s the only thing that really bothers me. And what also bothers me is that there are things that you can only do while walking.”

3) *Handling of and impacts from social environment*

Social impacts were reported in most cases (89.5%). An important aspect for many children and adolescents was social exclusion or worry about this. Examples included feeling sad when excluded from activities such as playing football or being bothered by insults or insensitive remarks, which sometimes happened unintentionally. However, acceptance and support from peers was also regularly mentioned, for example that friends naturally take one’s limitations into account when initiating activities. The interviews also highlighted that some patients appreciated being asked about their condition, while others preferred not to explain it at all or not repeatedly.

Mother of a boy, 7 years: “He has a somewhat special pronunciation. . people who don’t know him, who then don’t understand him, often make stupid comments. ‘Why do you talk so funny?’ Disparaging remarks that you make in passing. Probably not meant in a bad way by the other person, but which of course have a negative effect on the person concerned.”

Girl, 11 years: *“*So the Pompe disease doesn’t bother me that much, because I also have normal friends, they just accept me for who I am. . And we just play what I’m good at.”

4) *Plans and thoughts for the future*

For about two thirds of the children and adolescents (63.2%), statements were expressed indicating future-related thoughts about the disease. These encompassed two main aspects. Some thought about disease progression or early death (e.g., worrying that symptoms would get worse or hoping that they would remain stable), whereas others were troubled by impacts on long-term plans, such as difficulties studying abroad due to the ERT or giving up professional aspirations because of physical demands.

Mother of a boy, 6 years, with previous acute life-threatening episode: “There are days when he always tells me that he has to start looking for a woman to marry. . And then one day later he tells me that when he’s dead, we have to throw him in a hole and throw earth on top of him, because that’s how it’s done. That’s still so childlike, he still hasn’t really grasped it.”

Adolescent boy, 18 years: “I would actually have liked to join the army. . and that wasn’t possible because of my disease and the situation was so, so stupid.”

5) *Having a disease*

Experiences to so with having a disease were evaluatively categorized based on their valence. Some expressed a sense of acceptance: “having PD is okay”. Such neutral to positive experiences included handling the disease well overall, having gotten used to it, or feeling unique because of it, even though it can be quite annoying at times. In contrast, the disease was also reported as a burden: “having PD is quite burdensome.” These negative experiences included feelings such as intense sadness and anger about having it, a sense of inequality, and a desire to avoid any confrontation with the disease, including treatment appointments. Notably, 36.8% of cases reported both positive and negative statements, whereas 42.1% expressed only neutral to positive statements and 21.1% only negative statements.

Teenage boy, 13 years: “I don’t find it that bad because I’ve already adjusted to it and gotten used to it. So it’s actually not such a big issue.*”*

Mother of a teenage boy, 18 years: “He doesn’t want to be a patient. He wants to be normal. He wants to be like his peers.”

### Area III: experiences to do with treatment

Four main categories of treatment experiences were derived, each with up to four subcategories (see Table 4).

**Table 4:** Experiences to do with treatment: description of qualitatively derived categories

Main & Subcategories	Key points and examples
*Experience around…*	
1) ERT	
- ERT overall	• Accepting ERT as part of daily life: a thing that needs to be done. Still, attitudes ranged widely: - Neutral to positive experience (e.g., not minding it, grateful for it, neither liking nor disliking it, looking forward to it) - Ambivalent experience (e.g., bothered by some aspects, but appreciating others, very annoyed by it at times) - Negative experience (e.g. almost always angry, annoyed, and/or anxious on the infusion day or even the day before, not experiencing the ERT as beneficial) • Switch to home infusion: experiencing it as more positive or less negative (e.g., (very) grateful for the switch)
- Infusion and medical staff	• Afraid or bothered by the infusion due to fear of injection pain, worry whether the infusion is being carried out properly^a^, or discomfort with hospital setting (e.g., due to atmosphere) • Not very bothered by the infusion (e.g., never afraid or becoming less over time)^b^ • Switch to home infusion: experiencing the infusion as less burdensome due to good relationships with the consistently same nursing team and greater trust in their administration of infusions
- Impact on daily life	• Bothered by the frequency or length of ERT appointments impacting daily life (e.g., leading to missed leisure activities or school, boring or exhausting journeys to hospital, impossibility to go on longer vacations) • Not bothered by it or appraising it as a positive impact on daily life (e.g., not minding missing school, enjoying playing with the home nurse or video games during the infusion) • Switch to home infusion: experiencing the impact on daily life as less negative or more positive (e.g., less time required, more flexibility in rearranging appointments, more freedom to do what you want during infusions) • Switch to infusion at school: experiences ranged from preferring it over home infusion (e.g., even better incorporated into life) to bothersome (e.g., worried to stand out because of it)
- Port infections^c^	• Experiences of port infections included feeling unwell due to the infection, increased injection-related pain, fear of surgery, and increased fear of infusions for weeks up to months afterwards
2) Medical check-ups (no subcategories)	• Overall attitudes ranged from neutral/positive (e.g., not minding them) to ambivalent (e.g., seen as important but very exhausting) to very negative (e.g., scary, annoying, unnecessary) • Experiences to do with examinations ranged from not minding them^d^ (e.g., finding performance tests motivating) to being very bothered (e.g., very afraid of blood draws^e^) • Experiences to do with other impacts ranged from enjoyable (e.g., fun day-trip with parents), to annoyed, exhausted, or burdened by travel, missed activities, or having to stay overnight at the hospital^f^
3) Supplementary therapies (no subcategories)	• Positively experienced aspects to do with physio- or equine therapy, logopedics, etc. including good relationships with therapists, group therapies with peers, or experiencing some therapies or exercises as playful or beneficial • Negatively experienced aspects included finding some therapies or exercises too exhausting or not beneficial, feeling burdened by additional time commitments^g^, or disliking all disease-related therapies (e.g., preferring normal fitness workouts over physiotherapy)
4) Other treatment-related issues	
- Medical aids	• Experiences to do with breathing devices, mobility devices, etc. including having become grateful for it, knowing that it is required, but not appraising it positively, or being bothered by it because of dependency, required planning (e.g., not all places accessible for mobility devices), discomfort (e.g., orthoses), or seeing it as a symbol of the disease (e.g., port)
- Other topics	• Bothered by the reimbursement process (e.g., concerns that the insurance might stop covering an increased enzyme dosage or the home infusion) • Feeling disappointed or reassured by potential developments regarding new therapies

Note

a-e: Potential factors influencing certain experiences around treatment are listed (based on participants’ remarks and/or coder observations):

^a^ Fear of injection pain or worry about the execution of infusions may increase due to previous experiences in hospitals (e.g., lack of familiarity with port injections, no time for applying EMLA cream)

^b^ Fear of injection pain or worry about the execution of infusions may decrease or disappear with age and/or transition to home infusion

^c^ Port infection experiences may be especially relevant to children, potentially due to fewer adolescents having a port

^d^ Examination experiences may be influenced by the medical staffs’ communication, with openness and transparency preferred, and by repeated or single negative experiences (e.g., examinations performed without sufficient caution)

^e^ Fear of blood draws included in the check-ups may be increased because they are often performed through the vein instead of the port in children with ports

^f^ Check-up burden may be reduced by having a say in the organization (e.g., option for outpatient examinations at local specialists if possible, or if inpatient is necessary, option to stay in a hotel)]

^g^ Time burden may be reduced by having supplementary therapies at home or school

1. *ERT*

In all cases (100%), experiences to do with the ERT were discussed, and children and adolescents generally acknowledged it to be a necessary part of their lives. However, attitudes towards ERT varied, from considering it the greatest burden of the disease over ambivalence to even positive anticipation: “looking forward to it.” Infusion-related burdens were frequently mentioned, with some finding them very challenging and others having no issues. In daily life, some were very bothered about the frequency or duration of the ERT sessions, while others viewed them more favorably. Another topic was the switch of ERT from hospital to home. In general, this change was very well received due to the decreased impact on daily life and the presence of a familiar team of nurses whom they could trust with the infusion process.

Teenage boy, 18 years: “Nowadays, I don’t have many problems with the therapy. . In the past, I had to have it at the hospital. Then, it was very annoying to fit it into my daily life.”​.

Mother of a girl, 3 years: “So I think the infusion at home is great; for my daughter, it’s more like a friend coming to visit.”​.

2) *Medical check-ups*

Medical check-ups were also discussed in all cases (100%), expressing diverse attitudes from dislike through mixed feelings to not minding them. For some, blood draws were very burdensome, especially if performed through the vein instead of the port catheter, and the importance of how medical staff handled examinations was emphasized. In addition, some found the disruption of daily life annoying, especially for check-ups lasting more than one day, while others experienced part of check-up days as quality time with their parents.

Mother of a boy, 7 years: “Oh, if it wasn’t for the blood draw, it wouldn’t be so dramatic. Although he didn’t make a face during the last blood test. . Drawing blood isn’t really his thing because they do it via the vein and not via the port catheter. . It always depends a bit on which of the trainee doctors is on duty at the time. . more or less empathetic in relation to children. . But does he experience the entire hospital time as negative, no. Because as I said, we spend the night in the hotel beforehand, and we stop at McDonalds. What more could you want?”

3) *Supplementary therapies*

Experiences to do with supplementary therapies were mentioned in 73.7% of cases. While some enjoyed them as playful or helpful experiences, others considered them an additional time burden, not beneficial, or simply disliked any activities related to “having a disease.”

Father of a boy, 7 years: “At early intervention, it was very playful. . .That wasn’t really an issue. And apart from that he gets therapeutic riding. . He doesn’t want to go there, but when he’s there, he has fun. . But he generally doesn’t like these appointments. Because I think he always associates it with. . I only have to do it because I’m different from others.”

4) *Other treatment-related issues*

In about half of the cases (63.2%), various treatment-related issues were discussed, particularly experiences to do with medical aids (47.4%). Some expressed a sense of gratitude for their aids, whereas others were bothered by them for reasons such as dependency or viewing them as a symbol of disease. Other topics included concerns about insurance coverage for ERT and engagement with potential therapeutic advances, accompanied by feelings of either hope or disappointment.

Father of a teenage boy, 13 years: “At first it was with resistance. ‘I’m not wearing the mask, I’m not stupid.’ And now, as I said, it’s completely clear to him that ‘I need the mask.’. . Similar to the orthosis, lot of resistance at first: ‘I won’t put it on, it looks like shit.’ And now he says, well, ‘I’ll put it on, because if I wear it for longer, I notice that it helps.’”.

### Area IV: general emotional well-being/burden

Although the interviews did not explicitly inquire about general emotional well-being/burden, nearly all cases (94.7%) made statements about this. These statements were categorized evaluatively to determine whether they indicated that a child was generally not emotionally burdened or that he or she was, either intermittently or in general (see Table 5). Interestingly, higher levels of physical disability did not necessarily correspond to higher emotional burden. For most children and adolescents, including the most physically impaired, statements ranged only from no emotional burden to good general well-being (78.95%). Conversely, the two patients with clear emotional burden had relatively mild restrictions. In addition, statements were mixed for one adolescent.

**Table 5:** General emotional well-being/burden: description of qualitatively derived categories

Main & Subcategories	Key points and examples
1) General emotional well-being/burden	
- Not emotionally burdened	• Generally not burdened (e.g., not often unhappy resp. not more than peers, mostly even-tempered) to very happy in life (e.g., a lot of zest for life, having a happy childhood, appreciating life even more because of PD)
- Emotionally burdened	• Very emotionally burdened at times (e.g., sporadically having severe emotional slumps) to generally very unhappy (e.g., very often frustrated and miserable because of PD)

Mother of a severely disabled teenage girl, 14 years: “In her condition, what a joy for life she has. . she is actually never sad or moody, very rarely actually. Only when she’s in pain, otherwise, she is a friendly, cheerful girl.”​.

Teenage girl, 11 years: “I’m actually only angry when my mom forbids me to do something that I actually want to do. Or sad when something sad has happened or something like that. But otherwise, I’m actually mostly happy.”

Mother of a boy, comparatively mildly restricted, 10 years: “You think everything is actually fine, but then he has these slumps again, these psychological ones, he goes from being elated to being desperately sad and everything is bad.”

## Discussion

This study offers a broad picture of topics reported by children and adolescents with PD and their parents about how they perceive symptoms and related limitations (FDH-related aspects), how they experience the biopsychosocial impact of PD and its treatment on their lives (HRQoL-related aspects), and their general emotional well-being/burden. Through thematic content analysis of qualitative interviews with patients and caregivers, we identified 16 main categories, many with subcategories, relevant to PD-specific FDH or HRQoL. Our results advance the understanding of the burdens and needs of children and adolescents with PD and provide a basis for improving patient support, clinical care, and patients’ HRQoL. In addition, they are critical for ensuring the content validity of the first disease-specific questionnaire for pediatric patients with PD assessing both FDH and HRQoL.

In Area I, disease-specific FDH, broad heterogeneity was observed in perceived symptoms, their severity, and associated limitations among children and adolescents with PD, corresponding with the well-documented PD phenotypic variance [10]. Our sample depicted this variance, ranging from a female adolescent unable to move at all and dependent on constant ventilatory support to a male adolescent who considered himself physically fitter than most of his healthy peers. Muscle weakness in legs, arms, and core was the most prevalent symptom, leading to walking difficulties in almost all cases. Breathing difficulties, musculoskeletal issues, and fatigue were each problematic for about one third of participants, with other symptoms such as swallowing difficulties commonly reported. The issues mentioned and their heterogeneity align with qualitative studies investigating adults with LOPD [31] and are essential to developing and phrasing appropriate FDH-related items for our questionnaire.

Participants reported continuous or sudden improvements after starting ERT or adjusting dosages. In line with previous research [41, 42], accounts of symptom deterioration after several years of treatment were also identified. These findings emphasize the need of regular monitoring and the importance of the ongoing developments of new treatment options [43, 44].

In Area II, belonging to HRQoL, specific physical aspects were often a major burden for the subgroups of patients impacted by them. Intense pain in particular caused severe suffering. This is in line with a systematic review of 48 qualitative studies on children and adolescents with neurological or musculoskeletal disorders which indicated that pain is a main cause of reduced quality of life [45]. Regular assessment and adequate treatment of pain is therefore important in PD patients. Given the interplay of biological, psychological, and social factors contributing to chronic pain, multidisciplinary treatment such as a combination of psychological, physical, and medical interventions is recommended for pediatric pain management [46]. Physical limitations emerged as a key impact, leading to negative emotions such as frustration in almost all patients, even those with comparatively mild restrictions. This was probably because not only the restrictions themselves but also their social consequences were bothersome. This aligns with reports from ambulatory boys with Duchenne muscular dystrophy that the inability to keep up with peers is especially relevant to them [47]. Although physical limitations cannot be directly changed, the social exclusion associated with them could be addressed, for example by school-based interventions. Studies focusing on awareness enhancement have shown promising results in improved attitudes and acceptance of peers with disabilities [48, 49].

The appraisal of having *a disease* as either “okay” or “quite burdensome” was also important. Cognitive and emotional perceptions and appraisals of illness have long been discussed as central factors influencing coping and psychological adjustment to chronic disease in patients of all ages [50–52]. In our sample, “having PD” was viewed exclusively negatively by the two patients who were emotionally very burdened. Furthermore, an overall negative view of PD overlapped with other negative experiences related to being confronted with the disease: disability-related shame, annoyance at being asked about PD, and all treatment-related appointments. To challenge dysfunctional illness-related cognitions and coping strategies and improve psychological well-being, cognitive behavioral therapy has been proposed for pediatric patients with physical diseases [53].

In Area III, also HRQoL, patients acknowledged the necessity of treatment, although some found it very burdensome, as with other chronic conditions [45]. Consistent with a previous qualitative study investigating hospital-based ERT in which some participants expressed strong wishes for home-based infusion [32], the transition to home infusion generally increased treatment satisfaction by reducing both infusion-related burden and disruptions to daily life. Given the huge relief it brings and its safety [54, 55], ERT should be available at home for all patients.

Medical check-ups were experienced as scary, annoying, or even unnecessary by some of the patients. Thus, and in line with general recommendations for pediatric patients with chronic conditions, medical teams should carefully consider the necessity and frequency of follow-ups [12]. Furthermore, our findings suggest that open communication about the aims of examinations and patient involvement in organizing check-ups, for instance deciding between outpatient or inpatient between visits, could reduce burden. To reduce injection-related burden from both ERT and check-ups, a combination of topical anesthetics, age-appropriated distraction, and positioning for comfort is recommended for pediatric patients [56].

In Area IV, the main finding was that most patients in our sample, including the most severely affected, were not generally emotionally burdened (Area IV). This suggests that, overall, most pediatric patients adapt well to their condition, and that severe limitations do not necessarily lead to lower emotional well-being. Similar phenomena, known as the “disability paradox,” have been described in other chronic diseases [57]. A study on adults with PD also found no significant correlation between functional disability and mental health [58]. Thus, the emotional well-being of pediatric patients should be assessed independently of objective disease severity to identify those who may benefit from psychological support.

Overall, our results highlight that not only physical but also various psychosocial factors impact children and adolescents with PD. However, current recommendations on the clinical management of pediatric patients with PD do not yet incorporate such aspects [59, 60]. Therefore, it is important that forthcoming recommendations take this into account by mirroring approaches undertaken for children with similar diseases such as spinal muscular atrophy [61].

The main limitation of our study is the use of a convenience sample, which may introduce selection bias and thus limit the generalizability of the results. In this respect, it should be noted that children and adolescents with severe health issues who could not follow the study procedures had to be excluded, resulting in less self-reported interview data from more affected patients. To address this, we included parents regardless of their child’s health status. Additionally, the cross-cultural generalizability of our findings may be limited because we only included participants with sufficient German knowledge from three German-speaking countries. Furthermore, although the clear separation of FDH from HRQoL was a strength, this dual focus resulted in a lengthy interview manual with many questions. Thus, some restrictions on follow-up questions were necessary to manage acceptable interview duration, which in turn potentially limited the depth of exploration of some topics. Future qualitative studies focusing on narrower constructs with more representative samples would enhance understanding of the unique challenges faced by children and adolescents with PD. In addition, while comparable with other qualitative studies devised for questionnaire development [27, 31, 62], our sample size is relatively small for quantitative estimations. Therefore, caution is advised when interpreting frequency data. Also, the qualitative design and sample size precluded rigorous identification of potential risk and protection factors. Although the present results suggest that objective disease characteristics are poor indicators of HRQoL in pediatric patients in PD, future quantitative studies with larger samples are needed to test the impact of medical variables on HRQoL and to assess potentially influential sociodemographic factors such as age and gender.

An important strength of this study is the clear distinction between FDH and HRQoL, which addressed past and ongoing inconsistencies in the literature [15, 16, 38]. In addition, the international patient recruitment resulted in a diverse sample and reduced center-related bias. The use of a structured interview manual based on previous experience [26, 27] and expert input ensured consistency in data collection, and the content analysis was performed using established procedures and software. Finally, a qualitative approach such as ours enhances content validity when developing a questionnaire [24, 25] and provides broad in-depth insights into the disease-specific health perceptions and experiences of children and adolescents with PD.

## Conclusion

Our qualitative interview study identified key physical and psychosocial challenges faced by children and adolescents with PD. Interestingly, many young patients adapted well to their condition in their overall emotional well-being. However, the various impacts of the disease and its treatment were also linked to substantial burdens that were not proportional to objective disease severity. Some of these can probably be alleviated, for example through tailored psychosocial support. Therefore, it is crucial to identify patients at risk for impaired HRQoL. Our newly developed questionnaire, informed by the present results, should be tested as a screening tool for this purpose. Additionally, our results underscore the importance to patients of administering ERT at home.

## Data Availability

Interview data are not publicly available due to their sensitive nature. The data that support the findings of this study are available on reasonable request from the corresponding author.
